# Atypical endocrine manifestations in Gordon syndrome caused by CUL3 mutation: a case report

**DOI:** 10.3389/fmed.2026.1862901

**Published:** 2026-07-20

**Authors:** Mahsa Fatahichegeni, Mohammad Amin Ansarian, Hongjun Lv, Jiao Fu

**Affiliations:** Department of Endocrinology and Metabolism, The First Affiliated Hospital of Xi'an Jiaotong University, Xi'an, Shaanxi, China

**Keywords:** hyperkalemia, hypertension, insulin resistance, pseudohypoaldosteronism type II, testicular hypoplasia

## Abstract

Gordon syndrome (Pseudohypoaldosteronism type II) is a rare autosomal dominant disorder characterized by hyperkalemia, hypertension, and metabolic acidosis. Among the four causative genes, *CUL3* mutations produce the most severe phenotype, yet endocrine manifestations beyond growth delay remain poorly described. We report a 22-year-old male who presented with chronic hyperkalemia, hypertension, insulin resistance with steatohepatitis, and testicular hypoplasia with elevated gonadotropins, consistent with compensated primary testicular dysfunction. Genetic analysis identified a *de novo* heterozygous *CUL3* c.1207-26A>G splice-site mutation resulting in exon 9 skipping. Treatment with hydrochlorothiazide normalized blood pressure and serum potassium while improving metabolic and hormonal abnormalities. Notably, these improvements reversed upon treatment discontinuation. This case suggests that certain endocrine manifestations in *CUL3*-related Gordon syndrome may be secondary to chronic electrolyte imbalance rather than direct genetic effects, highlighting the importance of comprehensive endocrine evaluation and sustained thiazide therapy in affected patients.

## 1 Introduction

Gordon syndrome, also known as Pseudohypoaldosteronism type II (PHA II), is a rare autosomal dominant renal tubular disorder characterized by hyperkalemia, hyperchloremic metabolic acidosis, low plasma renin activity, and salt-sensitive hypertension. Since its first clinical description in the 1960s, advances in molecular genetics have identified four major causative genes: WNK1, WNK4, KLHL3, and CUL3, all of which are key regulators of the WNK-SPAK/OSR1-NCC signaling cascade. This pathway modulates the activity of the thiazide-sensitive sodium-chloride cotransporter (NCC) in the distal convoluted tubule and plays a central role in maintaining sodium and potassium homeostasis (1). The hallmark hyperkalemia in Gordon syndrome stems from impaired renal potassium excretion, mainly due to NCC overactivation, which enhances sodium-chloride reabsorption. This leads to volume expansion and suppression of the renin-angiotensin-aldosterone system (RAAS), resulting in relative hypoaldosteronism (2, 3).

Beyond suppressed RAAS, tubular aldosterone resistance also plays a critical role. Reduced sodium delivery to the aldosterone-sensitive distal nephron diminishes electrogenic sodium reabsorption through the ENaC (Epithelial sodium channel), impairing potassium secretion via ROMK (renal outer medullary potassium) channels. Moreover, WNK4 and CUL3 mutations may directly inhibit ROMK function, exacerbating potassium retention (4). Collectively, impaired aldosterone production and tubular responsiveness create a physiological mimic of aldosterone deficiency, underpinning the pseudohypoaldosteronism phenotype.

Among the four genetic variants, CUL3 mutation is known to produce the most severe phenotype. CUL3 encodes Cullin 3, a scaffold protein that forms a cullin–RING E3 ubiquitin ligase complex responsible for targeted protein degradation, including WNK kinases. Pathogenic mutations in CUL3, particularly splice-site mutations resulting in exon 9 skipping, impair the proper degradation of WNK kinases, leading to the constitutive activation of NCC and, consequently, marked salt retention, hyperkalemia, and hypertension (5).

Patients with CUL3-related PHA II often present at an earlier age and with more pronounced biochemical derangements than patients with WNK or KLHL3 mutations. Additionally, extra-renal manifestations such as growth retardation, intellectual disability, and neurodevelopmental disorders have been increasingly recognized in patients with CUL3 mutations, expanding the known phenotypic spectrum beyond electrolyte imbalance alone (5). Despite growing recognition of CU3′s systemic effects, other endocrine abnormalities, particularly those related to insulin sensitivity and reproductive function, have rarely been reported in PHA II.

In this context, we present a unique case of a young adult male with a *de novo* CUL3 mutation, who developed not only classical features of Gordon syndrome but also reversible insulin resistance and primary testicular failure. Notably, both metabolic and gonadal parameters improved significantly with thiazide diuretic therapy and relapsed upon its discontinuation. To our knowledge, this is the first report to document such endocrine manifestations in a genetically confirmed CUL3-related Gordon syndrome, suggesting that chronic electrolyte disturbances may contribute to broader metabolic and reproductive dysregulation. This case expands the clinical scope of CUL3-related disease, indicating that an expanded endocrine evaluation may be warranted in similar patients.

## 2 Case presentation

A 22-year-old Chinese male was referred to our center in October 2023 with a four-year history of persistent hyperkalemia and poorly controlled hypertension. The patient first exhibited elevated serum potassium levels (ranging from 5.0 to 7.0 mmol/L) during a routine health screening at age 18. Despite normal renal function, potassium levels remained elevated. 1 year later, at age 19, he was diagnosed with hypertension (blood pressure of 170/100 mmHg). Treatment with carvedilol was initiated but failed to adequately control his blood pressure or correct the electrolyte abnormalities.

In August 2023, the patient was admitted to a local hospital for further evaluation. He was found to have hyperchloremic metabolic acidosis and insulin resistance with steatohepatitis. Hormonal testing revealed low-normal serum testosterone levels with elevated LH and FSH, consistent with primary testicular dysfunction. Scrotal ultrasonography showed bilateral testicular hypoplasia with reduced testicular volume (left: 31 × 20 × 15 mm; right: 23 × 14 × 13 mm). A 3 mm pituitary microadenoma was found on MRI, and hormone evaluations—including ACTH, cortisol, TSH, prolactin, and growth hormone—were all normal, suggesting a non-functioning adenoma. His pubertal milestones included voice deepening and the development of a prominent Adam's apple around age 13. Karyotype analysis showed a 46, XY chromosomal pattern. The patient had undergone two sessions of extracorporeal shock wave lithotripsy in previous years for nephrolithiasis. There was a family history of hypertension (father at age 40, grandmother at age 70), but no family history of type 2 diabetes or metabolic syndrome.

The patient, with a height of 162 cm and a weight of 50 kg (BMI = 19.1 kg/m^2^), was referred to our hospital on October 13, 2023. On admission, his blood pressure was 170/100 mmHg, and serum potassium was elevated to 5.59 mmol/L. Serum sodium and chloride concentrations were 144.2 mmol/L and 108.8 mmol/L, respectively. Venous blood gas analysis revealed a pH of 7.42. In addition, a reduced carbon dioxide combining capacity (18.8 mmol/L) and an elevated anion gap (25.5 mmol/L) were identified, suggesting the presence of acid–base disturbance (Table 1). Hormonal assessment revealed suppressed plasma renin activity at 0.20 ng/ml/h (normal: 0.25–5.82) and low-normal aldosterone levels at 122.92 pg/ml (normal: 70–300), confirming the characteristic pattern of relative hypoaldosteronism despite significant hyperkalemia (Table 1). Hepatic steatosis was confirmed by transient elastography (CAP score: 282dB/m) and elevated transaminases (ALT 135U/L and AST 72U/L). His fasting glucose level was normal at 4.87 mmol/L, but his fasting insulin was elevated at 31.2 μIU/ml. The calculated HOMA-IR (Homeostasis Model Assessment of Insulin Resistance) was 6.75, indicating insulin resistance. Re-evaluation of his hormonal profile again showed elevated LH and FSH with low-normal total testosterone (Table 1). Interestingly, semen analysis revealed normal sperm count (71.98 million), motility (78.85%), and forward progression (47.58%), indicating preserved spermatogenesis despite testicular hypoplasia. Unfortunately, additional endocrine parameters such as sex hormone-binding globulin (SHBG), free testosterone, and inflammatory markers were not assessed.

**Table 1:** Serum electrolytes, hormones, and other biochemical parameters.

Parameter	On admission	HZT 12.5 mg bid		HZT 6.25 mg bid	HZT withdraw	HZT re-initiation 6.25 mg bid		Normal range
		1 month	6 month	3 month	1 month	3 month	6 month	
Blood pressure and serum lab tests								
Blood pressure (mmHg)	170/100	110/70	90/60	125/80	156/95	122/80	124/78	120/80
Na^+^ (mmol/L)	144.2	140.6	142.1	141.2	144.8	144.0	144.9	137–147
K^+^ (mmol/L)	5.59	4.38	4.54	5.18	5.51	4.19	5.16	3.5–5.3
Cl^−^ (mmol/L)	108.8	98.6	97.5	103.8	107.3	102.1	107.1	99–110
CO_2_ binding rate (mmol/L)	18.8	25.3	26.6	23.3	20.7	24.4	20.9	22–29
Anion gap (mmol/L)	25.5	–	–	–	–	–	–	8–16
Aldosterone (pg/ml)	122.92	–	154.71	150.1	233.66	–	196.16	70–300
Renin (ng/ml/h)	0.20	–	5.79	2.61	1.31	–	3.41	0.25–5.82
ALT (U/L)	135	–	113	37	23	46	40	9–50
AST (U/L)	72	–	56	24	20	24	41	15–40
Fasting insulin (μIU/ml)	31.2	27.72	26.85	12.52	30.87	–	12.93	6–27
Fasting glucose (mmol/L)	4.87	4.46	4.17	4.00	4.04	3.85	3.74	3.9–6.1
HOMA-IR	6.75	5.49	4.98	2.23	5.57	–	2.15	–
LH (mIU/ml)	11.520	–	10.150	9.9	11.3	–	14.3	1.700–8.600
FSH (mIU/ml)	13.650	–	10.290	10.44	11.25	–	14.2	1.5–12.4
Testosterone (nmol/L)	13.210	–	17.510	12.87	16.11	–	13.8	8.64–29
Pituitary prolactin (ng/ml)	21.23	–	18.66	13.55	–	–	11.4	4.04–15.20
Urine tests								
24 h potassium (mmol)	22.72	–	23.09	39.01	22.4	–	84.55	25–125
24 h chlorine (mmol)	97.72	–	106.20	142.8	176.0	–	399.51	110–250
24 h calcium (mmol)	6.39	–	1.10	1.46	4.77	–	3.22	2.5–7.5
24 h magnesium (mmol)	2.02	–	2.52	1.47	3.89	–	4.77	3.0–5.0
24 h phosphorus (mmol)	13.44	–	10.75	24.2	14.3	–	62.73	12.9–42.0

Laboratory parameters of the patient before and after hydrochlorothiazide (HZT) treatment. Values were measured at admission, after 1 and 6 months of HZT 12.5 mg twice daily, after 3 months of reduced HZT dose (6.25 mg twice daily), 1 month after HZT withdrawal, and 3 and 6 months of HZT 6.25 mg re-initiation. Parameters include blood electrolytes, liver enzymes, metabolic markers, hormones, and 24-h urinary excretion values. All measurements were performed under standardized conditions, with normal reference ranges provided.

**Figure 1:**
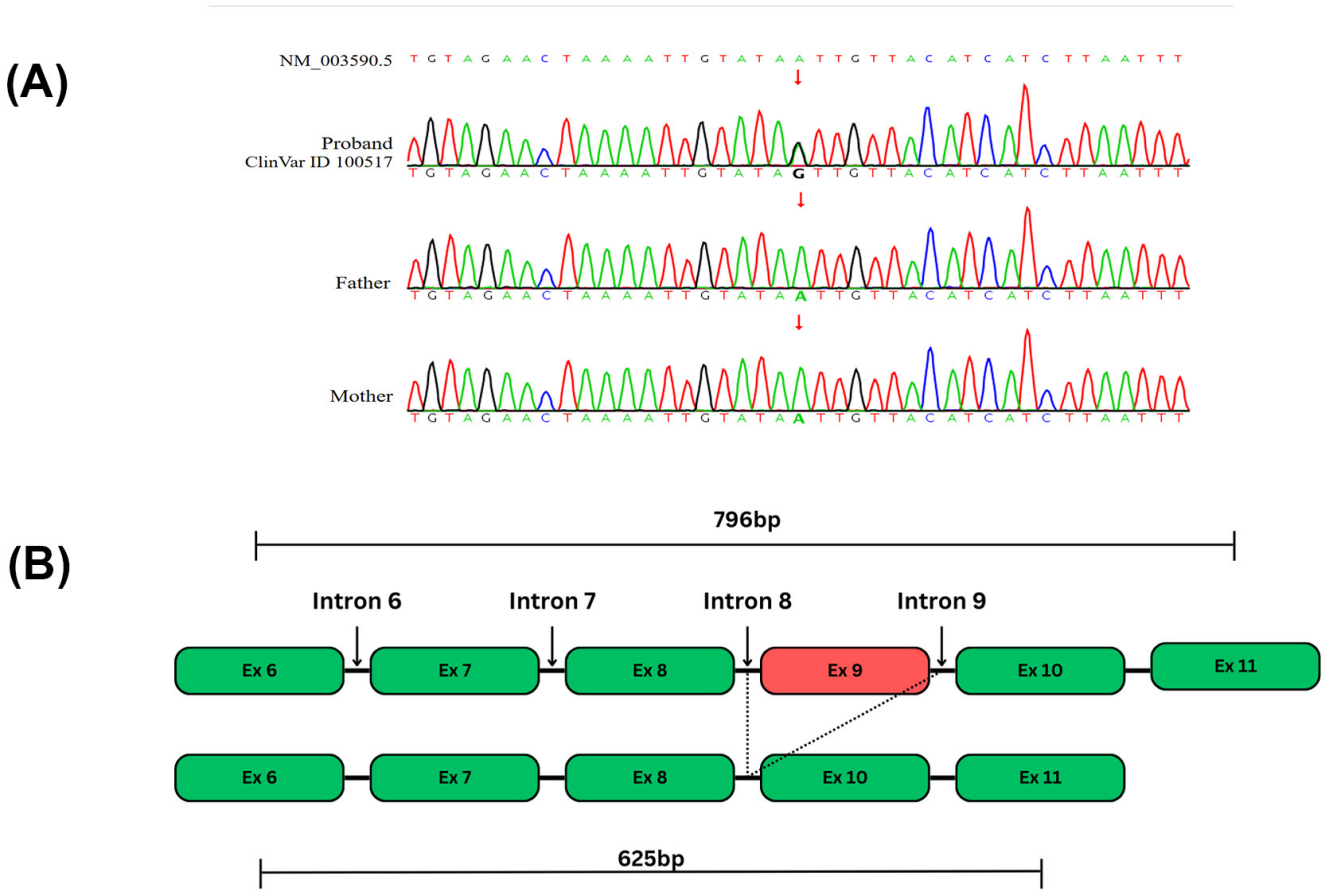
CUL3 mutation analysis and its effect on exon splicing. **(A)** DNA sequencing chromatograms showing the heterozygous c.1207-26A>G mutation in the proband (top) compared to wild-type sequences from both parents (middle and bottom), confirming *de novo* inheritance. Red arrows indicate the mutation site. **(B)** Schematic representation showing predicted exon 9 skipping resulting from the intronic splice site mutation, leading to altered CUL3 protein function. This mutation has been previously reported to cause Gordon syndrome through the disruption of normal CUL3 splicing patterns.

Before proceeding to genetic testing, a systematic differential diagnosis was performed for early-onset hypertension accompanied by persistent hyperkalemia and low renin activity. Conditions causing low-renin hypertension, including inherited disorders such as Liddle syndrome, apparent mineralocorticoid excess, and congenital adrenal enzyme deficiency, as well as acquired states of mineralocorticoid excess, were considered. However, these conditions are typically associated with hypokalemia due to enhanced distal sodium reabsorption and potassium excretion (6). The coexistence of hypertension, persistent hyperkalemia, low renin activity, and metabolic acidosis strongly suggested Gordon syndrome (pseudohypoaldosteronism type II). Therefore, whole-exome sequencing was performed and identified a heterozygous c.1207-26A>G intronic mutation in the CUL3 gene (Chr2:224503848; ClinVar ID: 100517). This variant is known to cause pathogenic exon 9 skipping and disrupts the WNK-SPAK-NCC signaling cascade involved in renal electrolyte transport (5). Genetic testing of the patient's parents confirmed the mutation was *de novo* (Figure 1). Variant interpretation followed ACMG guidelines and was supported by database cross-referencing (HPO, OMIM, GHR), confirming the variant's pathogenicity.

The patient was initiated on hydrochlorothiazide (HZT) at 12.5 mg twice daily. Within 1 month, his blood pressure normalized (110/70 mmHg), and serum potassium decreased to 4.38 mmol/L. Liver enzymes and fasting insulin levels improved substantially (Table 1). After 6 months, the HZT dose was tapered to 6.25 mg twice daily, with continued stability of blood pressure, electrolyte balance, liver function, and gonadotropin levels. Notably, there was no additional intervention targeting insulin resistance or hypogonadism beyond thiazide therapy.

One month after discontinuing HZT on the patient's own accord, his serum potassium rose again to 5.51 mmol/L, and blood pressure returned to hypertensive levels (156/95 mmHg). HZT was subsequently reintroduced at a dose of 6.25 mg twice daily. After 6 months of retreatment, blood pressure returned to normal, fasting insulin decreased to 12.93 μIU/ml, and HOMA-IR decreased to 2.15. Liver enzyme levels also improved, further supporting a temporal association between correction of electrolyte disturbances and recovery of metabolic abnormalities (Table 1).

## 3 Discussion

This case exemplifies the classical pathophysiological consequences of CUL3-mediated NCC dysregulation, while also revealing previously unreported endocrine manifestations that appear to arise secondary to chronic electrolyte disturbances. The close temporal association between electrolyte correction and endocrine improvement suggests a functional rather than a structural basis for these abnormalities.

As expected, the patient responded well to low-dose HZT, a direct NCC inhibitor, with normalization of serum potassium, blood pressure, and acid-base balance. This favorable response further supports the clinical diagnosis of Gordon syndrome (7).

In addition to the characteristic electrolyte abnormalities, this case presents two endocrine abnormalities not previously reported in CUL3 mutation carriers: insulin resistance with steatohepatitis and testicular hypoplasia accompanied by elevated gonadotropins. These abnormalities improved during HZT therapy and recurred on its discontinuation, suggesting a potential functional link between electrolyte disturbances and systemic endocrine dysfunction.

While CUL3 mutations have been associated with growth retardation and neurodevelopmental delay, comprehensive endocrine evaluation is rarely documented. For instance, in a systematic study by Hureaux et al., 71% of patients with exon 9 skipping exhibited growth failure, but no assessment of insulin or reproductive hormones was conducted (8). Previous reports have only speculated about “impaired reproductive fitness” in CUL3 mutation carriers (1), without documented clinical or biochemical evidence. Therefore, to our knowledge, this is the first report suggesting that endocrine abnormalities in CUL3-related Gordon syndrome may be both present and reversible.

The pivotal question is whether the metabolic abnormalities and gonadal dysfunction observed in this patient represent direct consequences of CUL3 dysfucntion or secondary manifestations of the chronic electrolyte disturbances characteristic of Gordon syndrome. Current evidence supports the possibility of both mechanisms. CUL3 is increasingly recognized as a regulator of cellular metabolism beyond its established role in renal electrolyte transport. Experimental studies have demonstrated that hepatic CUL3 deficiency can induce NRF2-dependent reductive stress, altered lipid metabolism, and systemic insulin resistance, suggesting that impaired CUL3 function itself may predispose to metabolic abnormalities (9). However, the rapid and reproducible improvement of insulin resistance following HZT treatment, its recurrence after treatment withdrawal, and subsequent improvement after treatment re-initiation strongly suggest that electrolyte correction played a dominant role in the metabolic changes observed in this patient.

Chronic electrolyte disturbances may contribute to metabolic dysfunction through several potential mechanisms. Hyperkalemia can acutely stimulate pancreatic β-cell depolarization and insulin secretion, potentially contributing to sustained hyperinsulinemia under chronic conditions. In parallel, metabolic acidosis has been shown to impair insulin signaling through mechanisms involving oxidative stress, inflammatory activation, and altered intracellular signaling pathways. Therefore, persistent hyperkalemia and acidosis may act synergistically to aggravate insulin resistance in susceptible individuals (10–12).

Regarding reproductive function, the patient exhibited reduced testicular volume with elevated gonadotropins but preserved serum testosterone levels and normal spermatogenesis, a phenotype more consistent with compensated primary testicular dysfunction rather than overt hypogonadism. Insulin resistance has been associated with impaired Leydig cell steroidogenic capacity and altered hypothalamic–pituitary–gonadal axis regulation (13). Therefore, the improvement in gonadotropin levels following correction of metabolic abnormalities raises the possibility that systemic metabolic disturbances contributed to the observed gonadal phenotype. Moreover, although CUL3 is expressed in testes and participates in spermatogenic processes (14, 15), the patient's preserved sperm parameters and the rapid gonadotropin normalization with HZT argue against a major defect in spermatogenesis. Nevertheless, a subtle direct effect of CUL3 dysfunction on Leydig cell development or steroidogenesis cannot be completely excluded and warrants further investigation.

Although CUL3 mutations cause severe, multisystem Gordon syndrome (5), and hepatic CUL3 deletion in mice can induce NRF2-driven insulin resistance (9), these findings stand in contrast to the rapid reversibility of endocrine dysfunction with simple electrolyte correction in our patient. Proteomic studies confirm broad cellular disruptions from CUL3 variants (5), potentially affecting endocrine cells. However, the threshold at which correcting electrolytes improves the abnormalities suggests that any direct effects of the mutation on insulin sensitivity or Leydig cell function are subordinate to the dominant pathophysiological influence of the electrolyte disturbances.

### 3.1 Study limitations

This report reflects a single patient's clinical trajectory and cannot be generalized. We did not perform molecular functional studies to confirm the effect of the CUL3 variant on protein function or NCC activation. The absence of key biochemical markers, including SHBG, free testosterone, inflammatory markers, and dynamic endocrine testing, further limits our ability to characterize the mechanisms underlying the hormonal abnormalities. No preserved serum samples were available for retrospective assessment. These parameters will be incorporated into future follow-up evaluations.

Additionally, the reversibility of metabolic and gonadal changes was observed over a relatively short time frame. Long-term sustainability of these improvements remains unknown and should be addressed through extended follow-up in future studies.

### 3.2 Clinical implications and future directions

This case highlights the potential for systemic metabolic and reproductive dysfunction secondary to chronic electrolyte disturbances in CUL3-related Gordon syndrome. However, given the presence of confounding factors and the limited scope of investigation, these findings should be interpreted as hypothesis-generating rather than conclusive.

Future efforts should focus on:

- Cohort studies evaluating endocrine abnormalities in CUL3 mutation carriers.
- Mechanistic studies to clarify how electrolyte derangements affect insulin and gonadal function.
- Functional genomics to directly assess how CUL3 mutations affect β-cell and Leydig cell physiology.
- Longitudinal studies tracking hormonal responses to thiazide therapy over extended periods.

## 4 Conclusions

This case expands the potential phenotypic spectrum of CUL3-related Gordon syndrome to include reversible endocrine disturbances. However, causality cannot be established. The metabolic and reproductive findings observed here may represent secondary consequences of chronic electrolyte imbalance rather than direct genetic effects. Clinicians should be aware of the potential for systemic involvement in patients with CUL3 mutations, and further research is needed to elucidate the prevalence, mechanisms, and clinical implications of these findings.

## Data Availability

The original contributions presented in the study are included in the article/supplementary material, further inquiries can be directed to the corresponding authors.
